# Limitations of the Karnofsky Performance Status Scale in kidney transplant recipients

**DOI:** 10.1080/07853890.2022.2068806

**Published:** 2022-05-09

**Authors:** Ahmed Mahmoud Osman Daoud, Mohamed Khalaf, Mahmoud Nassar

**Affiliations:** a Nephrology Division, Internal Medicine Department, Kasr Alainy Faculty of Medicine, Cairo University, Cairo, Egypt; b Gastroenterology Division, Internal Medicine Department, Medical University of South Carolina (MUSC), Charleston, SC, USA; c Internal Medicine Department, Icahn School of Medicine at Mount Sinai/NYC H+H Queens, New York, NY, USA

Dear Editor,

We have read with great interest the manuscript written by Ali et al. regarding the impact of kidney transplantation on functional capacity (FC) [1]. The authors used the Karnofsky Performance Status Scale (KPS) to assess the FC of kidney transplant recipients (KTRs). Since the 1950s, the KPS has been used as one of the earliest assessment tools. Users assign individuals a score between 0 (dead) and 100 (active, no restrictions) based on their ability to carry out daily activities. KPS was developed to assess patients with cancer. Currently, it is widely used to assess functional abilities both pre- and post-transplantation. The Organ Procurement and Transplantation Network (OPTN) requires reporting of KPS results at the time of transplantation for all adult recipients as a surrogate for measuring frailty.

The KPS facilitates the evaluation of functional ability. However, this assessment is dependent on the individual’s perception of his/her own abilities, which may not be reproducible. Furthermore, the process of categorizing symptoms as mild or more significant and making decisions about which symptoms to regard as concerning are not clearly described and are primarily left to the individual who assigns a score. Given the variability in user reporting, validity and reliability are questioned. A study of the mean KPS scores across transplant programs by researchers with the Scientific Registry of Transplant Recipients (SRTR) highlighted this drawback [2].

Frailty is prevalent among both dialysis and predialysis patients [3,4]. As reported by McAdams-DeMarco et al., frailty after transplantation is likely dynamic in kidney transplant (KT) recipients of all ages. In the first month post-KT, frailty deteriorates and then improves by three months post-KT [5]. Renal transplant recipients who were frail at the time of KT had significantly higher frailty scores in the long run. However, they had a greater likelihood of improvement in their Physical Frailty Phenotype score (PFP) and physiological reserve compared to non-frail renal transplant recipients, suggesting that KT may be beneficial even for frail candidates and that frailty is not an irreversible state of a poor physiological reserve for all KT recipients. The improvement in physiologic reserve may improve physical and kidney disease-specific quality of life among frail KT recipients. However, not all of these improvements are sustained over time [5].

## Data Availability

Data sharing is not applicable to this article as no new data were created or analysed in this study.
